# The varying rationality of weakness of the will: an empirical investigation and its challenges for a unified theory of rationality

**DOI:** 10.1007/s11229-022-03807-7

**Published:** 2022-08-26

**Authors:** Michael Messerli, Julian Fink, Kevin Reuter

**Affiliations:** 1 Department of Philosophy, University of Zurich, Zollikerstrasse 117, 8008 Zurich, Switzerland; 2 Department of Philosophy, University of Bayreuth, 95440 Bayreuth, Germany; 3 Department of Philosophy, University of Zurich, Zürichbergstrasse 43, 8044 Zurich, Switzerland

**Keywords:** Weakness of will, Akrasia, (Ir-)rationality, Coherence-based rationality, Reasons-based rationality, Success-based rationality, Empirical study

## Abstract

Weakness of the will remains a perplexing issue. Though philosophers have made substantial progress in homing in on what counts as a weak will, there is little agreement on whether weakness of the will is irrational, and if so, why. In this paper, we take an empirical approach towards the rationality of weakness of the will. After introducing the philosophical debate, we present the results of an empirical study that reveals that people take a “dual sensitivity”, as we shall put it, towards assessing the rationality of weak-willed behavior. Put succinctly, intending *X* against your value judgements is assessed irrational; yet, in the same situation, intending *X* is assessed significantly less irrational if you judge *X* as something you ought to do. After discussing this result, we turn to the question of whether there is a plausible theory of rationality than can account for the dual sensitivity of the rationality assessments. We show that a success-based account can make sense of the dual sensitivity our empirical results reveal.

## Introduction

Two conceptions of weakness of will enjoy substantial support in the philosophical literature. Historically dominant has been the akratic or Davidsonian conception, according to which agents are weak-willed if, and only if, they intend (or intentionally act) against their all-things-considered value judgement (Davidson, 1970). In contrast, the resolution view, championed by Holton (1999), states that a person is weak-willed if, and only if, that person “revises an intention as a result of a reconsideration that they should not have performed.” (Holton 1999, p. 6)^1^ In order to illustrate both conceptions, let us consider the following scenario that also serves as one of the test cases for our empirical study (the vignette is an adapted version from May and Holton (2012)).

**Pastry** Imagine that you are worried about your weight. Your doctor has told you that you need to lose weight. Otherwise you might have some health problems in the future. In light of this, you think it’s best to go on a diet and plan to do so. You stock up on healthier food and buy a book on how to lose weight. It’s a few days into your diet, and you are at work chewing on a carrot. However, one of your co-workers brings in a large box of your favourite pastries from the local bakery, and you just love them.

What will you do? If you choose to stick to your diet, although you think it best to eat the pastry (all-things considered), then you act akratically. You are also akratic if you choose to eat the pastry, despite judging that sticking to your diet is best (all-things considered). In both cases, you intentionally act against your all-things considered value judgement. In contrast, the resolution view identifies weakness of will in the revision of resolutions. Consequently, if you eat the pastry in spite of your resolution, you act in a weak-willed manner, regardless of which option you value the most.

Although some scholars have been skeptical of the existence of akrasia, by now, the consensus seems to be that people indeed behave at times akratically, and at times irresolutely.^2^ The empirical results we report in this paper can be read as providing additional support for the existence of both the akratic and the irresolute type of weakness of will, but we will not discuss this point in any detail. We will also not quibble about which conception of weakness of will best fits our ordinary talk about weakness of will; several empirical studies have already investigated the folk notion of weakness of will [c.f., (Mele, 2010; May & Holton, 2012; Beebe, 2013; Rosas et al.,, 2018)]. The results of these studies suggest that laypeople seem to attribute weakness of will both to the violation of value judgement and the violation of a resolution (Holton, 1999); for a critical reply, see, e.g., (Johnson, 2019).

Instead, our main focus will be on investigating the *rationality* of weakness of will. Perhaps unsurprisingly, many authors have argued that akratic behavior is irrational. Davidson, for example, claimed: “What is wrong is that the [akratic] man acts, and judges, *irrationally*, for this is surely what we must say of a man who goes against his own best judgement” (ibid, p. 42, our italics). However, not everybody agrees with Davidson that acting against one’s all-things considered value judgement is *always* irrational. Cases have been discussed in which akratic intentions would hardly be considered irrational (c.f., Audi (1990); McIntyre (2006); Arpaly (2000)). We find a similar situation when it comes to violations of resolutions. While (Holton, 1999) indicates that it would not be rational to revise a resolution, (McIntyre, 2006) dissects the various forms of rationality that are at stake in resolution-violating intentions. On the one hand, so she argues, people always “deserve criticism for their failure to be resolute” (2006, p. 309)—a failure she states to be a procedural defect of rationality. On the other hand, violating a resolution might indeed be considered the rational course of action, if it turns out that there are reasons that outweigh the upholding of the resolution.

While philosophical discussions on the rationality of weakness of will have certainly been illuminating and highly creative in conjuring up interesting cases to consider, they do suffer from an important defect. So far, claims as to whether an akratic and/or irresolute agent lacks rationality, are supported by the intuitions and conceptual analyses of individual authors only. While their theoretical reasoning might be reliable, we have good reasons to be cautious of jumping too quickly to conclusions. In particular, the last two decades have seen a swath of challenges to the reliability and representativeness of philosophers’ intuitions (Machery et al., 2004; Swain et al., 2008). We therefore submit that akratic and irresolute forms of weakness of the will considered “ir/rational” will be important for building a robust theory regarding the rationality of weakness of the will. In addition, an empirical investigation will allow us to examine how akrasia and irresoluteness relate towards each other. For it might turn out that one of these phenomena is assessed as a far more serious transgression of the rationality than violations of the other norm.

Finally, there is another important reason for collecting rationality assessments of a large number of people (N = 807). Only by measuring the various factors that influence rationality assessments, can we evaluate whether any of the existing theories of rationality can provide a unified theory of rationality. In particular, as we show in the paper, our empirical investigation reveals an unexpected and intriguing “dual sensitivity”, as we shall put it, of the rationality assessments. The degree to which akratic intentions are assessed irrational depends on how an agent relates to the content of that intention. Our hypothesis is that it depends on whether the intention is seen by the agent as something she ought to do. We also suppose that if a theory of rationality is unable to make sense of this result, then this casts doubt towards the correctness of that theory.

This paper is organized as follows. Section 2 presents our empirical findings concerning the irrationality of weak-willed decisions. We designed two test cases and asked our study participants to evaluate the proposed options and to make a hypothetical decision. After that, we asked them to rate the rationality of their decisions. One significant finding is that the rationality assessment of weak-willed behavior critically depends on the content of the intention that contravenes one’s value judgement. Section 3 then turns to a theoretical assessment of the empirical results. We attempt to provide a theory of rationality that is able to represent the rationality assessments we gathered from the empirical study. Section 4 concludes the paper by discussing possible objections against our approach. We counter these objections both theoretically and experimentally, thereby also replicating the results of our main study.

## Empirical study on the rationality of weakness of will

The main aim of our empirical study is to analyze rationality assessments from responses to two scenarios that are designed to entice weakness of the will. We decided to collect data from a first-person perspective, asking subjects how they themselves would value choices and make decisions in situations in which they (are likely to be) torn between, for example, eating a pastry and keeping a diet.

While we could have also asked participants to assess the rationality of decisions of other people from a third-person perspective, such assessments are likely detached from the choices they would make themselves. They are detached, because the participants do not necessarily identify with the decisions of the putative protagonist. In contrast, if the participants indicate that they themselves would make, e.g., akratic decisions, rationality assessments directly tap into their own behavioral choices.^3^

In both “weak-will-enticing” scenarios, participants were asked to value two options, after which they were then asked to tell us how they would decide. We defined akratic decisions as those in which the decision did NOT match the person’s value judgement. Furthermore, we stipulate that our scenarios are offering a choice between a “deontic” and “non-deontic” option, as we shall put it. This distinction will become important when we will offer our explanation as to how the participants in our study rank the rationality of their choices. For this we will assume that in both scenarios one option (i.e., “sticking to the diet” and “writing an application letter”) is likely considered as the “deontic option”. That is, we will assume that on average people will consider this to be the option they ought to pursue.^4^ Moreover, in the Pastry case, the deontic option is the option that the person had made a resolution to uphold. In the Couch case, such a resolution was at most implicitly given (we will discuss this issue in 2.3).

### Methods

We recruited 522 participants using Amazon Mechanical Turk and paid a small fee for their participation. We randomly assigned each participant to one of the following two vignettes.^5^

**Pastry** Imagine that you are worried about your weight. Your doctor has told you that you need to lose weight. Otherwise you might have some health problems in the future. In light of this, you think it’s best to go on a diet and plan to do so. You stock up on healthier food and buy a book on how to lose weight. It’s a few days into your diet, and you are at work chewing on a carrot. However, one of your co-workers brings in a large box of your favourite pastries from the local bakery, and you just love them. All things considered, and independent of how you decide in the end, how do you value each of the two options:

- Refusing the pastry and stick to the diet.
- Eating a pastry and interrupt the diet.

**Couch** Imagine you are at home lying on the couch by yourself. Recently, an interesting job vacancy has been advertised in the newspaper. Getting that job would advance your career, but as part of the application package you need to write a 3-page motivation letter. You grab the remote control of your TV, zap through the channels and realize that one of your favorite movies starts right now. All things considered and independent of how you decide in the end, how do you value each of the two options:

- Switch off the TV and write the motivation letter.
- Watch the movie and postpone writing the motivation letter.

We presented the two options for both vignettes in a randomised order and asked the participants to rate each option’s value on an 11-point Likert scale, anchored at 0 (‘Not at all valuable’) and 10 (‘Extremely valuable’). After the participants rated both options, we then directed them to the next page with the following question:

**Decision Question:** You have just valued each of the two options. But how do you decide in the end? Please tell us what you will do:

For the pastry condition, we gave the participants two options (in a randomised order): (1) I refuse the pastry and stick to the diet. (2) I eat a pastry and interrupt the diet. For the couch condition, the options were: (1) I switch off the TV and write the motivation letter. (2) I watch the movie and postpone writing the motivation letter.

After the participants answered the second question, they then responded to the rationality question on the next page.

**Rationality Question:** Do you think you have made a rational choice?

Participants responded on a 7-point Likert scale, anchored at -3 (‘Not at all rational’) and 3 (‘Totally rational’).

### Results

We excluded 17 participants from further analysis as they indicated that English was not their native language. The remaining 505 participants identified as 244 females, 260 males, and 1 of non-binary gender. The mean age was $M_{age}$ = 36.61 years (SD = 11.47 years).

**Fig. 1:**
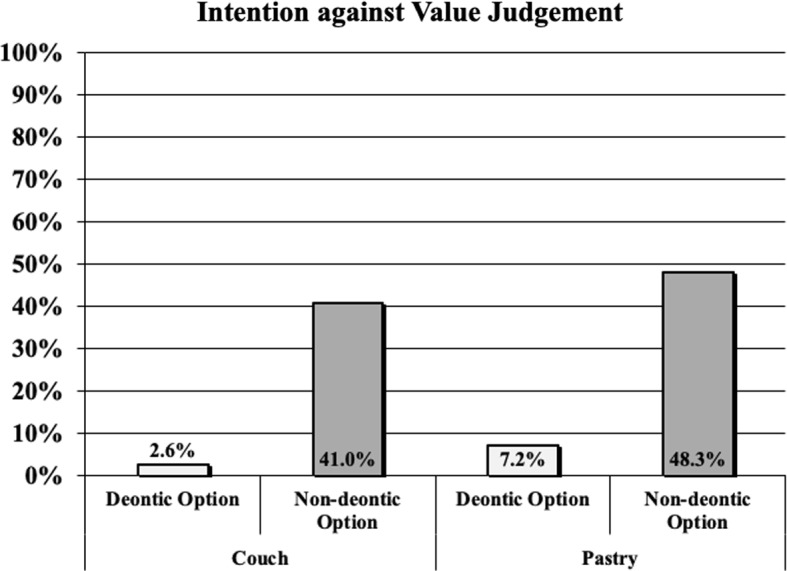
Amount of people in % whose intentions failed to match their all-things-considered value judgements (when the deontic option was chosen (white bars) and when the non-deontic option was chosen (grey bars) for both vignettes)

#### Intentions against value-judgements

Figure 1 displays the percentages of participants, whose intentions failed to match their value judgements, separately for each scenario. Almost half of those participants (54 out of 121) who chose the non-deontic option, indicated the deontic option to be more valuable. In contrast, only 19 out of 384 participants who decided in favor of the deontic option, reported a higher value for the non-deontic option.^6^ We used Pearson’s $\chi ^2$ test to detect a difference between the number of participants who violated their value judgement when intending the non-deontic option compared to the number of participants who chose the deontic option. The test revealed a significant difference between the non-deontic and the deontic condition: $\chi ^2 = 117.15; p < 0.001$. The results clearly show that significantly more people violate their value judgement when intending to act on a non-deontic, rather than a deontic option.

#### Rationality assessments

**Fig. 2:**
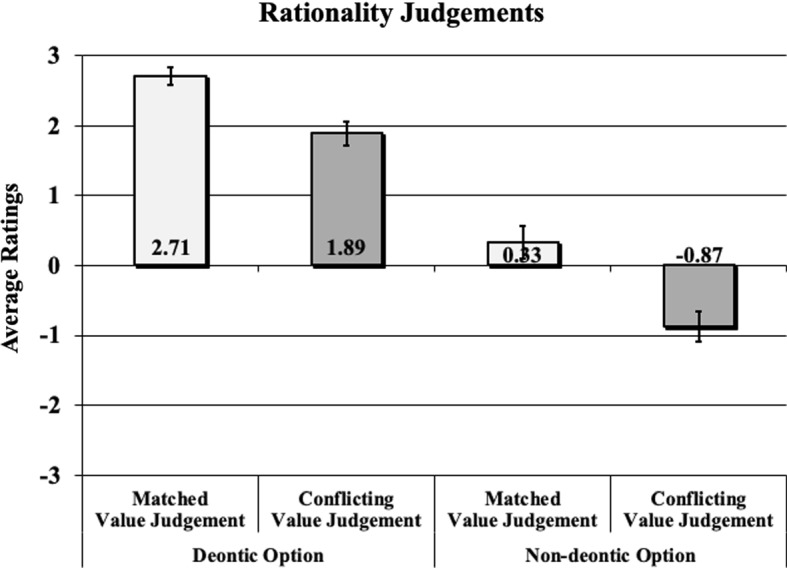
Mean responses to the rationality question in four different conditions. Error bars indicate standard error around the mean

To test the effect of people’s decisions on their rationality judgements, we conducted a 2 X 2 ANOVA with *Decision* (non-deontic vs. deontic) and *Akrasia* (akratic vs. non-akratic judgement) as independent factors, and *Rationality Assessment* as the dependent variable. *Decision* was a significant factor (F(3, 501) = 301.93, p < 0.001, $\eta ^2$ = 0.38), indicating that participants tend to attribute more irrationality when the agent chooses the non-deontic instead of the deontic option. There was also a main effect of *Akrasia* (F(3, 501) = 46.15, p < 0.001, $\eta ^2$ = 0.09), providing evidence that participants attribute more irrationality in cases where the decision contradicts the value judgment compared to cases where the decision matches the value judgment. No significant interaction was recorded between *Decision* and *Akrasia* (p = 0.192). All average ratings for the four conditions are displayed in Fig. 2.

### Discussion of the results

When comparing the responses across both vignettes, we found a significant difference between the percentage of participants (19 out of 384) who revealed an akratic tendency when intending to act on the deontic option, compared to the percentage of participants (54 out of 121) who displayed akrasia when intending to act on the non-deontic option). This result suggests that akratic behavior is a real and prevalent phenomenon.^7^ Importantly, the low number (2.6% and 7.2% for couch and pastry, respectively) of participants who intended against their value judgement when the intention was in favour of the deontic option, not only lends credence to the validity of our design, but also reveals that akratic responses primarily occur when people intend non-deontic options.

The data we collected in the pastry condition also indicates that a substantial amount of participants would behave irresolutely, i.e., eating the pastry despite having formed the intention not to give in to unhealthy food. Interestingly, the response profile was stable across the two different conditions (pastry vs. couch). This is quite remarkable insofar as the protagonist in the couch scenario has not formed an explicit resolution to write application letters when the opportunity arises. Thus, it might not be the resolution per se that influences people’s choices, but rather the intention to do what we believe we ought to do. In the next section, we will further elaborate on the role such an intention might play when making theoretical sense of our results.

In order to investigate the rationality of weakness of will, we asked the participants to self-assess the rationality of their answers. If akratic intentions are indeed irrational, we would expect that our participants consider themselves less rational when their value judgement conflicted with their intention. While our data revealed a significant effect of akrasia, the effect was rather small (effect size $\eta ^2$ = 0.09). When making a coherent choice for the non-deontic option, the average responses were rather neutral. When the participants made incoherent, non-deontic decisions, they showed a tendency to regard their decisions as irrational.^8^

We also recorded a rather large effect (effect size $\eta ^2$ = 0.38) of the deontic aspect on rationality judgements. Participants who intended the non-deontic option considered themselves to be less rational than their deontic favouring counterparts, even if the intention matched their value judgement. This suggests that deontic considerations have an even greater influence on rationality judgements compared to whether or not a person shows akratic behavior. These two empirically-grounded effects will form the background for the following theoretical discussion.^9^

## Theoretical investigation into the rationality of weakness of will

The participants of our study left us with an unequivocal ranking of the rationality of their purported decisions. How can we interpret this ranking from a theoretical perspective?

Figure 2 above shows the average rationality ratings of the (reported) intentions (decisions) in light of the value judgement. In a methodologically precautionary vein—and due to the relatively small N (=19) of ‘Deontic Intention & Conflicting Value Judgement—we will treat the average rationality values as merely ordinal degrees of rationality. Accordingly, Fig. 2 outlines the ranking of rationality among the following four situations:

*(Situation 1) Deontic-Intention & Matched Value-Judgement* 10

is more rational than

*(Situation 2) Deontic-Intention & Conflicting Value-Judgement*

which is more rational than

*(Situation 3) Non-Deontic-Intention & Matched Value-Judgement*

which is more rational than

*(Situation 4) Non-Deontic-Intention & Conflicting Value-Judgement*

Before we try to make theoretical sense of this rationality ranking, let us note some of its distinctive features. The two most striking observations are the following:

(1) Independent of whether switching one’s intention from a non-deontic to a deontic option makes one coherent or incoherent, one is *strictly more rational* by intending the deontic option than by intending the non-deontic option;

(2) Everything else being equal, one is *strictly more rational* by being attitudinally coherent (i.e. value judgement and decision are matching) than by being attitudinally incoherent (i.e., value judgement and intention are in conflict).

Consequently, rationality judgements seem to include a “dual sensitivity”, as we shall put it. First, rationality judgements are highly sensitive to deontic considerations. In our context, one’s rationality can always be improved by switching to the deontic option. Surprisingly, this holds true even if one assigns a higher value to the non-deontic rather than the deontic alternative. The rationality evaluations indicate that one would still be more rational by intending the deontic option over the non-deontic option.

Second, rationality assessments are also sensitive to the *coherence* of the intention and whether or not it stands in conflict with the value judgement (though in a weaker sense than the deontic status of one’s intention). Switching from attitudinal incoherence to coherence increases one’s rationality. However, this is conditional on not switching from a deontic to a non-deontic intention.

### Requirements of rationality

Does the dual sensitivity of observations (1) and (2) favour a particular theory of rationality? That is, is there a unified theory of rationality that can generate the rationality ranking and the involved dual sensitivity? Before we come to answer these questions, let us consider one way to deny the existence of a unified theory of rationality. One could suppose that the dual sensitivity itself already indicates that people operate with an ambiguous and non-unified notion of rationality. Thus, there is, on the one hand, one type of rationality one gains from aligning one’s intentions with what one believes one ought to do (this judgement identifies the deontic option). On the other hand, there is another type of rationality one gains from aligning one’s intention with one’s value judgement.^11^

No doubt, to distinguish these two types of rationality to the degree that they represent two distinct (and perhaps incompatible) concepts of rationality would be necessary if there was no single account of rationality under which these two uses could be subsumed under. But in order to establish if that is the case, we first need to explore if, in principle, there is a unified theory of rationality that can account for both types of rationality. Analogously, suppose we establish empirically that people sometimes use “morality” to refer to a consequentialist and sometimes to a deontological set of requirements. Then, as a first reaction, we would not suppose that people are operating with two distinct notions of morality here. Instead, we would try to reconcile both uses under one theory. (In fact, Tim Scanlon’s contractualist account of morality (Scanlon, 1998) can be seen as a case in point.) Of course, if there is sufficient evidence that it is impossible to draw up a plausible unified theory, then we would be required to resort to explain the dual use of morality by pointing at two distinct notions of morality. Thus, as announced, our task in this section is to see if a unified account of rationality can capture the dual sensitivity that emerged from our experiments.

Before we explore a unified theory of rationality, here is a brief remark about our methodology. Looking back at Fig. 2, we can see that our experiments generated a particular ranking of rationality among various options. More precisely, our experiments established a “is-more-rational-than” relation among Situations 1 to 4. However, most, if not all, contemporary theories of rationality (cf., e.g., Kolodny (2015); Broome (2013); Lord (2017), 2018; Kiesewetter (2017)) do not aim at establishing a “is-more-rational-than” relation (at least not directly) among various options. Instead, they aim at establishing the norms or demands of rationality, i.e., what rationality requires of us. For example, theories of rationality typically state that a person is required not to believe contradictions or to intend means necessary to one’s ends. But they are not primarily designed to designate when a person or situation is more rational than the other. In this sense, most contemporary theories of rationality are not directly designed to account for our empirical data directly.

Nevertheless, most theories of rationality are of course prepared to accept a conceptual or analytic nexus between one’s degree of rationality and the satisfaction and/or violation of requirements of rationality one is subject to (see, e.g., Broome 2013, p. 117). Accordingly, we will assume that, conceptually, rationality rankings stem from norms or requirements of rationality. That is, one’s degree of rationality must stem from a set of requirements one satisfies and/or violates. We will thus seek to identify which requirements of rationality would lead to the rationality ranking among Situations 1 to 4 that our experiments identified.

Here is a last brief methodological remark. In designing rational requirements that give rise to the rationality ranking among Situation 1 to 4, we will honor another theoretical constraint that we deem a conceptual matter of ir/rationality. We will assume that ir/rationality supervenes on the mind (Wedgwood 2002; Broome, 2013, 151-52; Cf. also Kiesewetter n.d.; for an opposite view, see Lord, 2017). That is, if one distinguishes the ir/rationality of a person in two different situations, then there must be a difference in the mental set-up of that person. This explains why we shall only postulate requirements of rationality that concern mental attitudes. Whether you satisfy or violate them will exclusively be a matter of (how you structure) your mental attitudes and will not depend on extra mental circumstances.

Let us now begin our task of formulating requirements of rationality with observation (2). All else being equal, you are strictly more rational by being attitudinally coherent than by being attitudinally incoherent. Hence, there must be a requirement that you satisfy when you switch from ‘conflicting value judgements’ to ‘matched value judgements’. We suggest the following requirement:

Best Intention Requirement (BIR): If you intend *X*, then you do not value any alternative option higher than *X*.^12^

Recall Situation 1-4. BIR suffices to explain why a person is more rational in Situation 1 than in Situation 2. In the former, one prefers the deontic option and abstains from evaluating the non-deontic option higher than the deontic option. In the latter, one intends the non-deontic option, but evaluates the deontic option higher than the non-deontic option. Hence, in Situation 1, you satisfy BIR, whereas you violate it in Situation 2. The same holds for Situations 3 and 4. BIR can thus account for the difference in the rationality ranking of Situations 1 and 2, as well as Situations 3 and 4.

However, BIR does not suffice to generate Fig. 2’s entire rationality ranking. One is more rational in Situation 2 than in 3, yet the former violates BIR while the latter satisfies it. As such, we need to propose another requirement of rationality in order to explain the rationality ranking between Situations 2 and 3.

On the face of it, this does not seem to be overly problematic. In Situations 1 and 2, one intends the deontic option. In Situations 3 and 4, one intends the non-deontic option. Consequently, a sufficient number of our survey participants took a decisively normative pro attitude towards intending the deontic option. The most straightforward version of this requirement reads as follows:

Normative Judgement Requirement (NJR): If you judge that you ought to *X*, then you intend to *X*.^13^

With BIR and NJR at hand, we are now in a strong position from which to explain the ordinal rationality ranking of Situations 1-4. Suppose you judge that you ought to pursue the deontic option. Accordingly, in Situation 1, you satisfy both NJR and BIR. In Situation 4, however, you violate both. Moreover, in Situation 2, you satisfy NJR while violating BIR—the reverse is true for Situation 3. Therefore, by simply including the assumption that satisfying NJR (rather than BIR) increases one’s degree of rationality, we can already fully explain the ordinal rationality ranking of Situations 1-4.

However, the postulation of NJR forces us to consider another requirement. BIR requires one to not value any alternative option higher than *X* if one intends *X*. NJR requires one to intend to *X* if one judges one ought to *X*. In a transitive spirit, it seems necessary (and plausible) to also assume the existence of a requirement not to value any alternative option higher than *X* if one judges one ought to *X*:

Best Judgement Requirement (BJR): If you judge that you ought to do *X*, then you do not value any alternative option higher than *X*.

With BIR, NJR and BJR at hand, we can now generate the rationality ranking of Situation 1-4 merely through the satisfying and violating of these three requirements. This is represented by the following diagram.

$$\begin{aligned} \begin{array}{lcc} &{}\quad \textit{Satisfy} &{}\quad \textit{Violate} \\ (Situation 1) &{}\quad BIR, NJR, BJR (3) &{}\quad - (0) \\ (Situation 2) &{}\quad NJR, BJR (2) &{}\quad BIR (1) \\ (Situation 3) &{}\quad BIR (1) &{}\quad NJR, BJR (2) \\ (Situation 4) &{}\quad - (0) &{} \quad BIR, NJR, BJR (3) \\ \end{array} \end{aligned}$$

Accordingly, in Situation 1, one satisfies 3 requirements and violates none. In Situation 2, one would satisfy 2 requirements and violate 1. In situation 3, one satisfies 1 requirement and violates 2. Lastly, in Situation 4, one satisfies no requirements and violates 3. Assuming that these requirements are sufficiently equal in weight, this then generates the ordinal rationality ranking of our empirical study.

### A unified theory of rationality

Thus far, we have articulated a set of rationality requirements that can explain the rationality ranking from Situation 1-4. However, can a unified theory of rationality explain these requirements?

We consider three contemporary theories of attitudinal rationality. The first locates attitudinal irrationality in being disposed to adjust a set of attitudes (Worsnip, 2018). The second understands irrationality as a necessary failure to respond correctly to reason (Lord, 2017; 2018; Kiesewetter, 2017). The third conceives of irrationality as the impossibility of the joint success of a set of attitudes and has been developed by one of the co-authors of this paper (Fink, forthcoming).

#### Worsnip’s dispositional theory

Let us start with considering Worsnip’s dispositional theory of ir/rationality (Worsnip, 2018). Accordingly, a set of attitudes is irrational if one is necessarily disposed towards abandoning at least one attitude once one becomes aware that they are held in conjunction. Or, in Worsnip’s own words (note that Worsnip uses the term ‘incoherent’ to identify an irrational set of attitudinal mental states):

“A set of attitudinal mental states is jointly [sic]^14^ incoherent iff it is (partially) constitutive of the mental states in question that, for any agent that holds these attitudes, the agent is disposed, when conditions of full transparency are met, to give up at least one of the attitudes.” (2018, p. 188)

Can this view help us to explain BIR, NJR and BJR? Let us focus on BIR. For this to work, people need to be universally disposed to align one’s intentions with one’s value judgements (or vice versa) under conditions of full transparency (i.e., being aware of your attitudes). That is, one must be disposed to avoid intending *X* while valuing *Y* higher than *X*, at least under fully transparent conditions.

However, the empirical part of this paper suggests the absence of such a disposition (see Fig. 1). Almost 50% of those who reported an intention for the non-deontic option did not seem reliably disposed towards aligning their (reported) intentions with their value judgements. We can thus assume that Worsnip’s theory does not adequately explain BIR, at least not as a strict requirement of rationality.^15^ Worsnip’s theory is, therefore, ill-equipped in that it cannot explain the rationality ranking of Situations 1-4.^16^

#### Lord’s and Kiesewetter’s reasons-based theory

We now examine another theory of attitudinal rationality. According to a reasons-based account, “[r]ationality consists in correctly responding to the objective normative reasons one possesses” (Lord 2017, p. 1121); see also Lord, 2018; Kiesewetter, 2017. Roughly, “possessing reasons” means that you have epistemic access to them. Accordingly, a pattern of attitudes is necessarily irrational if, and only if, the pattern guarantees a failure to respond correctly to the objective normative reasons one has epistemic access to. For example, a set of contradictory beliefs (i.e., a belief that p and a belief that not-p) is irrational, if and only if, necessarily, your normative reasons either require you not to believe p or they require not to believe not-p. By saying that “your normative reasons require you to *X*” we mean to say that if you do not *X*, you are committing a normative error (i.e., you are not entirely as you ought to be).

We doubt, however, that a reasons-based account of rationality can provide a unified explanation of BIR, NJR, and BJR. Particularly, a reasons-based explanation of BIR can lead to an implausible normative conflict when conjoined with a reasons-based explanation of NJR. We briefly demonstrate this below.

Let us first turn to BIR. It states that one is rationally required not to intend something if there is another option one values higher. If this requirement is to be covered by a reasons-based account of ir/rationality, then, whenever one judges one ought to *X* and values an alternative option higher than *X*, then one has an attitude that violates one’s available reasons. To guarantee this, a reasons-based explanation of BIR requires the truth of the following disjunction:

Disjunctive BIR (DBIR): Necessarily, either [your reasons require you not to intend *X*] or [your reasons require you not to value any alternative option higher than *X*].

That is, judging that you ought to *X* while valuing an alternative option higher than *X* must ensure that either:

(i) your intention that *X* violates what your reasons require of you; or

(ii) your valuing (an alternative option higher than *X*) violates what your reasons require of you.

Recall NJR: It requires that, if you judge that you ought to *X*, then you intend to *X*. Analogous to a reasons-based explanation of BIR, a reasons-based explanation of NJR requires the truth of the following disjunction:

Disjunctive NJR (DNJR): Necessarily, either [your reasons require you not to judge that you ought to *X*] or [your reasons require you to intend to *X*].

That is, judging that you ought to *X* while not intending to *X* must ensure that either:

(iii) your judgement (that you ought to *X*) violates what your reasons require of you; or

(iv) lacking an intention to *X* violates what your reasons require of you.

Consider now the following example. Suppose you are normatively permitted to judge that you ought to either *A* or *B*. In other words, you may judge that [*A* or *B*] is something that you ought to bring about. Due to the standard logic of permissions (according to which a permission implies the absence of a requirement), this implies that:

(3) It is *not* the case that your reasons require you not to judge that you ought to either *A* or *B*.

Let us apply (3) to DNJR. In the context of (3), DNJR says that:

(4) Necessarily, either your reasons require you not to judge that you ought to either *A* or *B*, or your reasons require you to intend either *A* or *B*.

Since (3) negates the left disjunct in (4) (i.e., your reasons require you not to judge that you ought to either *A* or *B*), (3) and (4) conjoined imply that:

(5) Your reasons require you to intend either *A* or *B*.

Suppose now that you are undecided about the comparative value of *A* and *B*. That is, you value neither *A* over *B*, nor vice versa. In addition, let us stipulate that you are in a situation where:

(6) You are normatively permitted to value *A* over *B*.

and

(7) You are also normatively permitted to value *B* over *A*.

In short, from a normative point of view, it is entirely up to you whether you value *A* or *B* higher; you are not required to value one over the other.

Let us look again at DBIR. In conjunction with (6), DBIR implies that

(8) Your reasons require you not to intend *B*.

This is because (6) implies that it is not the case that your reasons require you not to value some option over *B*; DBIR thus implies that your reasons require you not to intend *B*. Likewise, in conjunction with (7), DBIR implies that

(9) Your reasons require you not to intend *A*.

This is because (7) implies that it is not the case that your reasons require you not to value some option over *A*; DBIR thus implies that your reasons require you not to intend *A*.

However, contrast this with (5). That is, we have just established that DNJR implies:

(5) Your reasons require you to either intend *A* or *B*.

In short, a reasons-based explanation of BIR and NJR can lead to a substantial normative conflict. It can result in a situation where one’s reason requires one to *X* while simultaneously requiring one to not-*X*. This shows that a reasons-based view cannot adequately explain BIR and NJR. It must claim either that BIR and NJR are requirements of different types of rationality, or that one requirement is incorrect. Recall, however, that this would make it difficult (if not impossible) to explain the rationality ranking from Fig. 2.

#### Fink’s succcess-based theory

Let us now turn to a ‘success-based’ explanation of BIR, NJR and BJR. Accordingly, a set of attitudes is irrational if, and only if, these attitudes preclude the possibility of their joint success.

We suggest two distinct ways in which an attitude can succeed. First, an attitude can succeed in virtue of being correct. For example, a belief can succeed qua being true. Then the belief’s success is one of being correct. By contrast, an attitude can succeed in accomplishing its executive role(s). For example, an intention can succeed qua being realised. Then the intention’s success is one of fulfilling an essential causal aim (Fink, forthcoming)^17^

Can this view explain BIR, BJR and NJR? Let us first consider BIR and BJR. BIR states that rationality requires one not to intend *X* and to value another option higher than *X*. Analogously, BJR states that rationality requires one not to judge that you ought to *X* and to value another option higher than *X*.

Let ‘V(*Y*)> V(*X*)’ stand for that the value assigned to *Y* is higher than that assigned to *X*. Consequently, in order to explain BIR and BJR, there needs to be a sense in which combining

(i) an evaluate judgement: V(*Y*)> V(*X*), and (ii) an intention to *X* or judging that one ought to *X*

implies that one’s attitudes cannot jointly succeed.

We have already mentioned an *executive* success condition for intentions, namely realisation. However, intentions also have success conditions of *correctness*. Suppose, for example, that you became aware that *A* is incompatible with the, all-things-considered, best course of action available to you. We assume that this would dispose you not to intend to *A*. We further assume that this would also hold true for ought judgements. You would be disposed not to judge that you ought to *A*. We can thus consider this an essential disposition of an intention and ought judgement.

Suppose you intend or judge that you ought to *X*. Hence, a success condition for this intention would be that, all-things-considered, there is no option that is of a higher value than *X*.

Assuming that your evaluative judgement that *Y* is more positive than *X* is cognitive and belief-like, then truth would be one of its success conditions, i.e., all-things-considered: V(*Y* ) > V(*X*).

Under this interpretation, it would be unequivocally irrational to value *Y* over *X* while intending, or judging, that you ought to *X*. Your evaluation and intention, or ought judgement, preclude their mutual success. Your intention to *X* and your ought judgement to *X* succeed only if, all-things-considered, there is no option that is of a higher value than *X*. Your evaluative judgement that *Y* is of higher value than *X* succeeds only if the value of *Y* is higher than the value of *X*. Thus, the success of your attitudes would require, simultaneously, that there is no better option than X and that there is a better option than X. However, that is impossible. Your attitudes cannot succeed jointly.

A success-based account of irrationality can thus explain BIR and BJR. But what about NJR? Can a success-based account also explain this requirement? To do so, there must be a sense in which

(iii) an ought judgement that *X*, and (iv) the lack of an intention to *X*

implies that your attitudes cannot jointly succeed.

Suppose you judge that you ought to *X*. We assume that one success condition of your judgement is its realisation, i.e., you *X*. We assume that this holds true for both a cognitivist and non-cognitivist interpretation of ought judgements.^18^ However, the fact that *X* is a success condition for a judgement that you ought to *X* does not negate the possibility of an attitude’s success condition when combining (iii) and (v). The fact that you lack an intention to *X* does not necessarily mean that you will not end up *X*-ing. Indeed, you may *X* accidentally (i.e., without intending it).

However, simply applying a slight restriction to ought judgements will lead to a situation where (iii) and (iv) imply the impossibility of your attitudes’ success. Consider a case where you believe you ought to *X*, fail to intend to *X*, yet also believe that *X* will occur even if you do not intend to *X*. This does not seem to be an example of irrationality. By contrast, you are irrational only if your ought judgement refers to an *X* for which you also believe that you will *X* only if you intend to *X*.

Consequently, by restricting (iii) to ought judgements for which you also believe that X only if you intend to *X*, we are in fact dealing with a combination of three attitudes:

(iii) an ought judgement that *X*, (iv) the lack of an intention to *X*, and (v) a belief that *X* only if you intend to *X*.

With some rare exceptions, a belief that you ought to *X* will imply a belief that *X* only if you intend *X*. For example, suppose you believe you ought to study for an exam. It is almost certain that you will also believe that studying can only occur if you intend to do so. After all, studying for your exam will not happen accidentally. This implies that, for a large majority of judgements, attitude (v) is essential.

Conjoining (iii)-(v) puts us in a situation in which at least one of your attitudes will not succeed. Suppose it is true that *X* only if you intend to *X*. It follows that you can realise your ought judgement only if you intend to *X*. However, you do not intend to *X*, so your ought judgement ultimately fails to be successful. Alternatively, suppose it is not true that *X* only if you intend to *X*. Then you may realise *X* without intending to *X*, but then another attitude of yours lacks success, namely your belief that *X* only if you intend to *X*. In any case, one of your attitudes fails. NJR represents a credible requirement of rationality under a success-based explanation of attitudinal rationality.

The upshot is clear. The success-based account can explain why NJR, BIR, and BJR are correct requirements of rationality. It thus seems that the rationality verdict, and the resulting rationality ranking, tend to favour a success-based understanding of attitude-based ir/rationality.

## Summary and possible objections

### Summary of our investigations

In this paper, we raised two questions: How do people assess the irrationality of weakness of the will? Can we make theoretical sense of these assessments? Previous answers to these questions tended to remain within purely theoretical discussions. We deem this inadequate and thus conducted an empirical study on the rationality of weakness of will.

The participants’ average rationality assessments revealed a clear ranking of weak-willed intentions. People consider themselves less than fully rational when: (i) holding intentions that contradict their value judgements, and (ii) when intending a non-deontic instead of a deontic option. This result provides prima facie support for those who have argued for the irrationality of weakness of will. Moreover, those participants who intended the deontic option considered themselves rational well above the neutral point, even when their intentions contradicted their value judgements. In short, our data shows that akratic intentions are not fully rational and that we should consider those intentions less rational than intentions aligned to our value judgements.

In the theoretical part, we explored whether a unified theory of rationality can explain the average rationality ratings. The biggest challenge was to formulate a set of requirements of rationality that gives rise to the “dual sensitivity” of rationality judgements that our empirical investigation revealed. The dual sensitivity suggests that (i) we can always improve our rationality by coming to intend the deontic option (even if this goes against our value judgement). Moreover, (ii) we can always improve our rationality by coming to intend what we value more. Given our empirical data, we argued that the rationality ratings should be understood as resulting from a distinct set of requirements of rationality: BIR, NJR and BJR.

Moreover, we demonstrated that these requirements can neither be explained by a dispositional nor a reasons-based theory of rationality. Against the dispositional account, we posited that our empirical investigation reveals that people seem to lack a sufficiently reliable disposition to align their intentions and value judgements. Against reason-based accounts, we showed how such an account can end up facing implausible normative conflicts. Instead, we argued that a success-based theory of rationality can defend these requirements. That is, whenever you violate one of the stated requirements, the failure of (at least) one of your attitudes is guaranteed. This view not only explains the irrationality of failing to align one’s intentions with value judgements, but also shows why failing to intend the deontic option detracts from complete rationality.

### Possible objections

In the remainder of the paper, we tackle three objections against our findings.

#### Methodological skepticism

First, a methodological skeptic might question why the dual sensitivity we recorded should have any bearing upon theories of rationality? More specifically, one might object that the rationality assessments of laypeople should not guide our theorising about rationality—indeed, people often lack the competence to correctly assess their rationality. Moreover, one might hold that the folk concept of rationality is distinct from the concept of rationality within the field of philosophy.

However, both these points are highly questionable. According to the Corpus of Contemporary American English (COCA), the term ‘rational’ (16,909 hits) is roughly as frequent as the terms ‘generous’ (16,113 hits), ‘boring’ (17,925 hits), and ‘delicious’ (15,518 hits). Laypeople make frequent claims about a person’s rationality or behaviour, and should therefore be seen as competent users of the concept. At the very least, the burden of proof should lie with those who depreciate people’s competence to judge rationality. Should the objection rest instead on the idea that the folk concept of rationality is distinct from the technical concept used in philosophy, then it should be noted that those philosophers have engineered themselves away from what seems to be at the heart of philosophical investigation: the analysis of everyday concepts central to understanding ourselves and the world. At any rate, some philosophers of rationality have been explicit in stating that the best theory of rationality needs to align itself with our folk concept.^19^

#### Reproducing core findings

Second, in the previous section of our paper, we argued against Worsnip’s dispositional theory of ir/rationality. In response, one might claim that we have not sufficiently taken into account the “full transparency” aspect of the dispositional account.^20^ Worsnip writes:

“By “conditions of full transparency”, I mean conditions under which the agent knows, and explicitly and consciously believes, that she has the states in question, without self-deception, mental fragmentation, or any failure of self-knowledge (pertaining to those attitudes). Notice that it is not required for these conditions to be met that the agent acknowledge that her mental states violate a requirement as such. It is merely required that she acknowledge that she has the states that (perhaps unbeknownst to her) violate the requirement.” (2018, p. 188); original emphasis

The participants who took part in our experiment first gave their value judgement, after which they indicated the decision they would make. Arguably, some participants only became aware that they had the *two states in question* after they gave their responses. Hence, our empirical design might not satisfy Worsnip’s conditions of full transparency. In order to tackle this objection, we revised and reran our original experiment. Because the new experiment also responds to a further objection, we will first consider that objection and then present the results of that study.

The third objection highlights a possible confound of one of the scenarios we tested.^21^ In the Couch scenario, participants were told that they needed to decide between watching TV and writing a motivation letter for a job application. It was not clear from the vignette, however, whether they could have written the motivation letter at a later stage, e.g., the next day, because we had not specified a lingering deadline for the application. In fact, the inclusion of the word ‘postpone’ in one of the options of the value and decision question even suggests the possibility to write the letter later. If the participants indeed interpreted the Couch scenario along these lines, then our scenario would be more a case of procrastination rather than weakness of will.

We decided to address both these objections by running another empirical study. In order to respond to the full-transparency objection, we added a fourth question (after the value, decision, and rationality question). The participants were asked: “In light of your assessment of the previous questions, would you like to go back and either make a different decision or change the value ratings? Or do you stand by your initial judgements?” Participants had three possible response options: (1) I would like to go back and make a different decision; (2) I would like to go back and rate the value of the choices differently; (3) I stand by my judgements.

In order to avoid that participants interpreted the Couch scenario as a case of procrastination, we changed the wording of the vignette and presented the following scenario:

**Couch** Imagine it is 9 pm in the evening and you are at home lying on the couch by yourself. Recently, an interesting job vacancy has been advertised in the newspaper. Getting that job would advance your career, but you still need to write a 3-page motivation letter before the deadline at midnight. You grab the remote control of your TV, flip through the channels, and see that one of your favorite movies is starting.

The answer options were also changed compared to the original experiment to exclude the possibility to write the letter at a later stage. Thus, instead of “I watch the movie and postpone writing the motivation letter.”, the option now read “I watch the movie and don’t write the motivation letter.” A fourth question was added (see wording above). We recruited 302 participants via Prolific Academic.^22^ 15.2% (N=46) of the participants selected the response option “I watch the movie and don’t write the motivation letter.” Of those participants, 60.9% (N=28) gave a value rating that runs counter to their decision. Only three of them stated that they would like to go back and make a different decision. All others selected ‘I stand by my judgements.’ The rationality ratings for the four conditions were as follows:

- Deontic-Intention & Matched Value-Judgement: +2.43
- Deontic-Intention & Conflicting Value-Judgement: +0.50
- Non-Deontic-Intention & Matched Value-Judgement: −0.50
- Non-Deontic-Intention & Conflicting Value-Judgement: −1.71^23^

In response to Objection 2, the results demonstrate that hardly any participant would like to go back and make a different decision even after they had rated their previous responses to indicate a low degree of rationality. While this study confirms our previous results that participants who reported their intentions do not seem reliably disposed towards aligning their (reported) intentions with their value judgements, the outcome also shows that participants accept the discrepancy between intention and value judgement under full transpacency. Thus, Worsnip’s account seems to stand refuted by the data.

Our results also hardly provide any evidence that the data in our original study were partially driven by interpretations of procrastination (compare the third objection above). At least qualitatively, the results match those of the original study. The ordinal ranking of degrees of rationality were confirmed by the new empirical data. The outcome thereby also shows the robustness of our results in light of variations in the wording of the vignette as well as the recruitment of participants from a different platform.

In conclusion, given that the rationality ratings hold up to scrutiny, we can safely argue that our empirical findings indicate a profound preference for a success-based theory of rationality. A success-based theory gives rise to the requirements leading to the rationality ranking that our participants indicated. This suggests that rationality should be understood theoretically as having attitudes that can succeed jointly.

## Footnotes

1 There are other conceptions of weakness of will based on, for instance, passion (Jackson, 1984; Michael, 2019) and lack of self-control (Watson, 1977).

2 Hare (1952) argued that akrasia is conceptually impossible. According to him, value judgements guide our intentions and actions reliably. Davidson famously disagreed with Hare’s assessment. He argued that intending to *y* does not analytically imply an ‘all-things-considered judgement’ (see Davidson’s take on distinguishing between ‘all-things-considered’ and ‘unconditional evaluative’ judgements; Davidson (1970, pp. 37-41).

3 Third-person assessments might also suffer from being too evaluative and biased. That said, there are also disadvantages of using first-person reports. Participants might rationalize their own behavior or self-censor themselves.

4 Note that these deontic considerations are not necessarily reflected in people’s all-things- considered value judgements (“I value X more than Y, all in all.”). There is empirical evidence that people make decisions against their value judgements if deontic considerations are in play (Messerli & Reuter forthcoming). Also note that “deontic option” should be understood broadly here. It cannot be reduced to “morally best option”, for example. Instead, the idea is that people consider sticking to the diet or writing the letter as something they ought to do because, for example, this option has a greater long-term benefit, they have made a commitment, or it is socially accepted to do so. We leave it open what the exact content and source of the deontic judgment is.

5 The details of the study were preregistered on the Open Science Framework: https://osf.io/zksgf.

6 For the subsequent statistical analysis, we merged those responses in which participants decided in line with their value judgements with those (relatively few) responses in which the participants valued both options equally. We made this decision as people who believe both options to be equally valuable do not intend against their all-things-considered value judgement. Given that there was no significant effect of the type of scenario (couch or pastry) we used ($\chi ^2 = 0.66; p = 0.416$), we merged the data from both scenarios.

7 One might question, of course, whether it is likely that people will indeed behave in a way that is in line with the recorded responses. There is ample empirical evidence that the question of whether people’s responses are consistent with their real behavior depends on context (see, e.g., De Silva, 1991, Benz & Meier, 2008; and Lambooij et al., 2015).

8 Our findings also suggest that intentions that violate a previously formed resolution have a stronger effect on assessments of rationality compared to intentions that go against one’s value judgements. In other words, behaving irresolutely is deemed more irrational than being akratic. Of course, such a conclusion needs to be taken with some caution. First, only in the Pastry condition did we specify that the agent had formed a resolution (“In light of this, you think it is best to go on a diet and plan to do so.”) That the same pattern of results emerged in the Couch case, in which no explicit resolution was spelled out, is intriguing and warrants further study. Second, in order to make stronger claims about the comparative irrationality of akratic and irresolute intentions, a larger variety of scenarios needs to be investigated. Fortunately, the existing weakness of will literature is very rich in terms of scenarios and thought experiments, and we hope our research will motivate further empirical work on these cases.

9 The design of our study was not without problems. For instance, one might object that, although we asked the participants to provide us with their *all-things-considered* value judgements, they may have simply failed to reliably report them. Thus, it is possible that some participants considered the deontic option as more positive only in terms of furthering their overall goals, but in fact intended the non-deontic option because it reflected their all-things-considered value judgement. Note, however, that the participants consistently rated themselves as less rational when intending the option that violated their value judgement. The objection (i.e., participants did *not* give all-things-considered judgements that ran counter to their intentions) would only hold if they had not considered themselves less rational. Thus, the rationality assessments of the participants in our study produce evidence that the value question provides a suitable approximation for an all-things considered rating.

10 *Deontic-Intention* means that one intends the deontic option. *Matched Value-Judgement* means that intention and value judgement match. *Non-Deontic-Intention* means that one does not intend the deontic option. *Conflicting Value-Judgement* means that intention and value judgement do not match.

11 We are grateful to a reviewer of this journal for raising this point.

12 “Any alternative option” roughly refers to the actions you consider yourself able to perform by intending them.

13 In the debate on (structural) ir/rationality, the NJR is usually referred to as the “Enkratic Requirement” or “Enkrasia”. We would like to thank an anonymous reviewer for this. See, e.g., (Broome, 2013, Kiesewetter, 2017 and Worsnip, 2018). Most commonly, the “Enkratic Requirement” is (roughly) formulated as follows: “If you believe you ought to X, then you intend to X”. However, in order not to exclude non-cognitivist interpretations of this requirement (i.e., saying to yourself that you ought to *X* may not express a belief), we replace “believe” with “judge” and assume that a judgment that you ought to *X* may also express a non-cognitive attitude (such as a desire, plan or the acceptance of a norm, etc.).

14 It is the attitudes within the set that are jointly incoherent, not the set itself.

15 Note that Worsnip claims that the disposition in question must have considerable force. It must be such that the agent would (at least normally) “not [be] able to (or at least find it difficult to) sustain such combinations of attitudes under conditions of full transparency” (2018, p. 188).

16 Here is a quick objection to our argument. One may argue that our experimental design does not satisfy Worsnip’s condition of “full transparency”. That is, given the order of our questions, it is unclear whether our participants would perhaps not consider giving up one of their attitudes. We address this point with a second experiment in Section 4.2.

17 How can we identify conditions of attitudinal success? We suggest the following method: s is a necessary condition for attitude *A* to succeed qua correctness if, and only if, an essential disposition of *A* were to disappear on the realisation that s is not the case. Analogously, s is a necessary condition for attitude *A* to succeed qua fulfilling an essential executive role if, and only if, an essential disposition of *A* were to bring about *s*. For example, an essential disposition of a belief that *p* disappears on awareness of not-*p*; *p* is thus a success condition (qua correctness) of a belief that *p*. Likewise, an essential disposition of an intention to *a* is to cause *a*; *a* is thus a success condition (qua correctness) that *a* (Fink, forthcoming).

18 This may be easy to see within non-cognitivist interpretations of ought judgements. A non-cognitivist understanding of an ought judgement is to conceive of it as an intention. Since a key condition for an intention to succeed is realisation, the same then holds for an ought judgement. Matters tend to be more complicated regarding cognitivist interpretations of ought judgements. However, here an unrealised ought judgement implies a lack of success also. Suppose you judge that you ought, but fail, to *X*. If your judgement is true, then you failed to do something that you ought to have done. Your judgement is thus not entirely successful. If your judgement is false, then you failed in making a correct normative judgement, meaning your judgement was again not entirely successful. Hence, failing to do what you believe you ought to do entails that your judgement fails in some way.

19 Some evidence for this claim can be found in Kolodny (2005, pp. 515–517). Broome (Broome 2016, p. 6) also notes that “most philosophers who write about rationality intend to write about it as it is commonly understood”.

20 We thank a reviewer of this journal for raising this objection and suggesting a way for us to respond.

21 We are grateful to a reviewer of this journal for raising this objection.

22 The average age in the sample was 38.59 years with a gender representation of 151 male, 148 female, two indicating non-binary gender, and one abstaining from indicating their gender.

23 We conducted a 2 X 2 ANOVA with *Decision* (non-deontic vs. deontic) and *Akrasia* (akratic vs. non-akratic) as independent factors, and *Rationality Assessment* as the dependent variable. *Decision* was a significant factor (F(3, 298) = 42.03, p < 0.001, $\eta ^2$ = 0.12), indicating that participants tend to attribute less rationality when the agent chooses the non-deontic instead of the deontic option. There was also a main effect of *Akrasia* (F(3, 298) = 15.78, p < 0.001, $\eta ^2$ = 0.05), providing evidence that participants attribute less rationality in cases of a conflicting value judgment compared to cases of a matched value judgment. No significant interaction was recorded between *Decision* and *Akrasia* (p=0.357).
